# Implementation of a novel mHealth application for the management of people with diabetes and recently healed foot ulceration: A feasibility study

**DOI:** 10.1177/20552076221142103

**Published:** 2022-12-06

**Authors:** Samantha Haycocks, Rory Cameron, Mark Edge, Jayne Budd, Paul Chadwick

**Affiliations:** 1 Salford Care Organisation, 523611 Northern Care Alliance NHS Foundation Trust, Salford, UK; 2 Gendius Ltd, Cheshire, UK; 3 Royal College of Podiatry, London, UK

**Keywords:** Diabetes, diabetic foot ulcer, mobile application, patient engagement, recurrence, self-care

## Abstract

**Background:**

Diabetic foot ulcers (DFUs) cause significant morbidity and mortality. Faster referral to specialist clinics is associated with a reduced risk of severe DFUs. The INTELLIN^®^ diabetes management platform is a novel mHealth application for the management of recently healed DFUs and other complications, promoting engagement and expediting self-referral.

**Methods:**

To determine the acceptability, suitability, and usability of the INTELLIN^®^ platform, time until reulceration, site, ischemia, neuropathy, bacterial infection, and depth (SINBAD) score and incidence of self-referral for recurrence were assessed in patients from the Salford Royal NHS Foundation Trust. Patients and clinic staff also assessed platform usability. A Markov cost-utility model was used for the health economics and outcomes research analysis.

**Results:**

197 patients were assessed for eligibility and 15 entered the full analysis set (FAS). Through Week 52, 8/15 patients experienced recurrence, with a mean SINBAD score of 2.1 and mean duration of 2.6 days. Mean time to recurrence was 273.0 days (95% confidence interval 74.0, 484.0). No patients self-referred. Initial qualitative data showed high platform usability. The INTELLIN^®^ platform only required a relative reduction in recurrence of 5% versus standard of care (SoC) for an incremental cost effectiveness ratio of £20,000 per quality-adjusted life-year, suggesting potential for significant cost savings upon wider adoption. The barriers to enrollment encountered demonstrate the impact of socioeconomics on mHealth.

**Conclusions:**

These results suggest that the INTELLIN^®^ platform is required to provide only a small reduction in recurrence compared to SoC to be a cost-effective strategy for prevention of recurrent DFUs.

## Introduction

### Background

Diabetes is a chronic condition estimated to affect 4.6 million people in the UK.^1^ As the prevalence of diabetes is increasing,^1^ so too is the incidence of diabetes-related complications. A common microvascular complication of diabetes is neuropathy, which causes loss of pain and sensation in the extremities and can result in a diabetic foot ulcer (DFU).^2^ A DFU is a full-thickness wound, skin necrosis, or gangrene localized to an area of skin below the ankle.^3^ Risk factors for the development of a DFU include poor glycemic control over an extended period, peripheral arterial damage, and poor foot care.^2^

**Figure 1.**
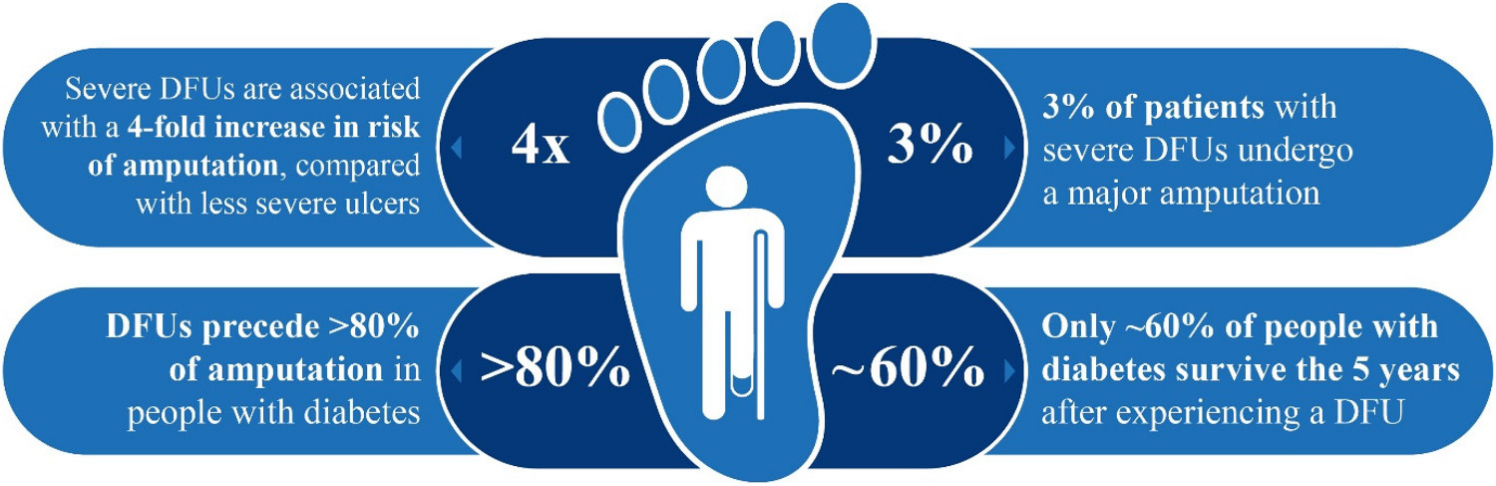
Morbidity and mortality associated with DFUs [figure adapted from^4, 5^].

Abbreviation: DFU, diabetic foot ulcer.

DFUs cause significant morbidity and mortality (Figure 1).^4, 5^ In a UK study, during the first year after a clinically infected index DFU, 15.1% of patients died, 17.4% had a lower extremity amputation, and 9.6% experienced a DFU recurrence.^6^ The often difficult and prolonged healing of DFUs, alongside the subsequent health risks, result in reduced quality of life for patients and substantial costs for healthcare systems. In addition, DFUs contribute to the current high UK National Health Service (NHS) burden, with incidence increasing by 93% between 2012–2013 (169,000 cases) and 2017–2018 (326,000 cases).^7^

As a result of the high patient burden and significant economic costs, there has been considerable focus on preventing DFUs and optimizing treatment in recent years. Optimal DFU preventative care includes the support of a multidisciplinary team, patient education and training, and faster referrals, most often achieved via self-referral.^8^ However, current best practice involving specialized foot clinics is costly and still dependent on patient self-care outside of the clinic. Faster and more appropriate referral to a specialist clinic after symptom onset has been linked to a reduced risk of patients developing severe DFUs and improved outcomes, as demonstrated by a study by Ndosi et al. in which patients with a clinically infected ulcer present for ≥2 months experienced a lower incidence of healing (hazard ratio 0.55; 95% confidence interval [CI] 0.39, 0.77).^6^

### INTELLIN^®^ diabetes management application background and aims

The INTELLIN^®^ diabetes management platform (Gendius Ltd, Macclesfield, UK) is a novel mHealth application for the management of patients who have experienced a recently healed DFU, among other diabetes complications, with bespoke software that promotes engagement with DFU care and expedites the self-referral process (Supplementary Figure 1). Focusing on the nine care processes (weight and body mass index [BMI]; blood pressure; glycated hemoglobin [HbA1c]; retinopathy screening; foot risk stratification; urinary albumin test; serum creatinine; smoking status; cholesterol levels)^9^ INTELLIN^^®^^ allows both patients and healthcare professionals to track and monitor their diabetes. Having a patient app and healthcare professional dashboard, data is shared giving a holistic view of someone's diabetes. During the development of INTELLIN^®^ user interviews were conducted to understand the needs, motivations, and expectations of an app for people with diabetes. The results of these interviews were used as the base for the app content (unpublished work). The patient enters data about their lifestyle and their diabetes while the healthcare professional can enter up to date results such as HbA1c, this bidirectional flow of data ensures both the patient and healthcare professional are always up to date with changes associated with diabetes. A list of the questions asked on the platform can be found in Supplementary Table 1. During the study, the platform supported the Podiatry team by giving diabetes management information directly to the team. This facilitated baroader approach to intervention or management. The team got an insight into whether the patient had had all their checks but also how they were managing their Blood Glucose, Blood Pressure, etc. Although this meant a more in-depth appointment that included checking the feet it also ensured the patient was managing their diabetes. The app also provides support and patient health education for the prevention and management of DFUs, with the aim of improving ulcer self-detection and to prompt individuals to take timely action in case of recurrence. Using algorithms, INTELLIN^^®^^, evaluates the entered data and determines the areas of concern for the patient to focus on, giving direction to clinically approved health information. For the patients on the study, this would focus on DFUs. Having this information at their fingertips allows the patient to make a quick reference should the condition of their feet change. Then entering the changes into the app would enable their healthcare professional to see the changes and contact the patient if necessary. Using INTELLIN^^®^^ does not replace the Diabetic Foot Screening program, it enhances it, by making patients aware of their priorities, providing up-to-date clinical information and clinician support. This unique approach promoted patient podiatrist engagement. The World Health Organization^10^ supports that patients who are engaged with their health care provider and better able to make informed decisions about their care while promoting the use of electronic tools to support this engagement.

Patients use the app in different ways, for example, a person with type 1 diabetes may use it daily, recording their blood glucose levels and activity, whereas a person with type 2 may only use the app to search for information as and when required or when a change in their health occurs. During the study, patients were encouraged to review their feet daily and enter changes when necessary; however, the clinical information was available 24 hr a day. Diabetes features as one of the priorities for care and quality outcomes in the NHS Long Term Plan and there is a significant potential value of such apps to help manage complications of diabetes, including DFUs.^11^

### Feasibility and cost-utility study aims

A feasibility study was carried out to assess the acceptability, suitability, and usability of the INTELLIN^®^ platform, as reported by patients and diabetic foot clinic staff. Aims were (a) to determine the acceptability, suitability, and usability of the INTELLIN^®^ platform using a 5-point scale, as assessed by (i) the patients and (ii) the clinical team; and (b) to assess the proportion of patients who self-refer for recurrence of DFU over a 12-month period, compared with the current average of approximately 40%.^12^ Socio-economic and patient demographic challenges that arose during the recruitment and enrollment process will be discussed, together with how such challenges may impact future mHealth studies. A health economics and outcomes research (HEOR) analysis was also performed to determine the level of clinical benefit required for INTELLIN^®^ to be cost-effective compared with standard of care (SoC), for the prevention and management of recurrent DFUs among people with diabetes at high risk of DFU in the UK.

## Methods

### Sample size

Data taken in the National Diabetes Foot Audit (NDFA) identified that Salford has around 33 new episodes of ulceration a month and around 55% are alive and ulcer free at twelve weeks. This indicated there would be potentially 15–18 people every month who could be eligible for the study. It was anticipated that, if recruitment was agreed at around 5 participants per month, recruitment would take 8 months, reviewed retrospectively for the first three months. 197 patients were assessed for eligibility, the main reason for non-participation was patients not having a smart phone (n = 58). Other reasons for non-participation included reulceration prior to the study start date (n = 28) and no contact following self-referral (n = 24) (Table 1).

**Table 1.** Baseline characteristics.

Baseline characteristic	Patients in the FAS (*n* = 15)
Diabetes mellitus	
T1DM, *n* (%)	3 (20)
T2DM, *n* (%)	12 (80)
Mean time from diabetes diagnosis, years (SD)	17.9 (10.1)
Most recent DFU prior to study entry	
Mean time since healing of last DFU, days (SD)	45.9 (23.9)
Mean (SD) SINBAD score of last DFU	2.1 (1.0)
Cases where last DFU was severe, *n* (%)	4 (27)
Patients receiving insulin, *n* (%)	6 (40)
Mean basal insulin dose per day, units (SD)	16.9 (20.1)
Mean bolus insulin dose per day, units (SD)	5.7 (10.0)
Number of patients overdue their annual 8 Care Processes, *n* (%)	2 (13.3)

Abbreviations: DFU, diabetic foot ulcer; FAS, full analysis set; SD, standard deviation; SINBAD, Site, Ischaemia, Neuropathy, Bacterial infection, and Depth; T1/2DM, type 1/2 diabetes mellitus.

### Patients

Patients were recruited from the Salford Royal NHS Foundation Trust (SRFT) catchment area, chosen for this study as the National Diabetes Footcare Audit (NDFA) identified that Salford had the potential for recruitment of eligible patients.^13^

To be eligible for inclusion, patients had to be ≥18 years of age, with a diagnosis of diabetes mellitus (type 1 or 2), documented HbA_1c_ >48 mmol/mol, the level defined for diagnosis,^9^ on their electronic patient record, and a history of a recent DFU (defined as being eligible for the NDFA and classified as healed for 4–12 weeks). Patients must also have provided informed consent, have no reasons that they could not be part of the study for 12 months, and have owned a smartphone on study enrollment.

Exclusion criteria included any comorbidity, for example, poor eyesight, which would limit the use of a smart phone, participation in a different interventional study within the last 30 days, and any critical illness that would prevent participation.

Patients were identified through self-presentation following advertisement across public forums, from within existing SRFT healthcare clinics, and from the contact information available as part of the NDFA.

### INTELLIN^®^ platform set-up and functionality

Patients completed a series of 36 questions to set their profile and medical history (Supplementary Table 1) within the mobile app, which were then used to populate and create the algorithms developed by the Advanced Data Analysis Centre at Nottingham University. Patients were able to input and track aspects of their diabetes management that might impact their foot health, including blood glucose levels, body mass index (BMI), blood pressure, and cholesterol levels. The platform also incorporated functionality, an SOS button, which enabled rapid self-referral to the SRFT foot care team. If triggered the SRFT foot care team would be alerted in order to contact the patient with an urgent appointment for review.

Additionally, the healthcare professional could input information into the web portal dashboard during clinic visits, which would then sync to the app. The platform also provided patients with daily tips concerning appropriate foot care and managing diabetes and diabetes-related complications.

### App data collection schedule

Follow-up visits were scheduled at Weeks 1, 4, 16, 28, 40, and 52. Time until reulceration was measured through Week 52, while the Site, Ischemia, Neuropathy, Bacterial Infection, and Depth (SINBAD) score was assessed at screening for the most recent DFU prior to study entry and at the time of referral for any instances of DFU recurrence. The SINBAD score is used routinely to document DFU severity, as recommended by National Institute for Health and Care Excellence (NICE) (Table 2).^5^ The proportion of patients who self-referred for recurrence during the study was also assessed.

**Table 2.** SINBAD scoring system for DFUs.

Category	Definition	SINBAD score
Site	Forefoot	0
	Midfoot and hindfoot	1
Ischemia	Pedal bloodflow intact: at least one pulse palpable	0
	Clinical evidence of reduced pedal blood flow	1
Neuropathy	Protective sensation intact	0
	Protective sensation lost	1
Bacterial infection	None	0
	Present	1
Area	Ulcer <1 cm^2^	0
	Ulcer ≥1 cm^2^	1
Depth	Ulcer confined to skin and subcutaneous tissue	0
	Ulcer reaching muscle, tendon, or deeper	1
Total possible score	-	6

Abbreviations: DFU, diabetic foot ulcer; SINBAD, Site, Ischemia, Neuropathy, Bacterial Infection, and Depth.

The annual check for the NHS 8 Care Processes was completed at baseline in all patients who did not have results recorded within the last 3 months. Measurement of the NHS 8 Care Processes was repeated upon any presentation of a recurrent DFU in patients who had been in the study for >6 weeks and at Week 52 in those patients who completed the study. The NHS 8 Care Processes include measurement of HbA_1c_, cholesterol (total, high-density lipoproteins, and low-density lipoproteins), serum creatinine, urine albumin (to calculate albumin:creatine ratio), blood pressure, BMI, smoking status, and foot examination, with the latter comprising visual assessment, vascular assessment (toe brachial pressure index and ankle brachial pressure index), and neurological assessment (detection of monofilaments).^14^ Although the NHS has recently adopted retinal screening as a ninth Care Process, this measure was not included in the present study as retinal screening is often performed by private establishments and so the results are not available on the NHS electronic patient record system.^15^

### INTELLIN^®^ usage during the study

Platform user experience was monitored using a questionnaire (Supplementary Tables 2A and 2B, for patients and clinicians, respectively) completed at Week 1, Week 16, and at the end of the study (either Week 52, early withdrawal visit, or at the DFU recurrence visit). Prior to each study visit, frequency of patient app usage was reviewed remotely by clinicians via the platform dashboard; the results were then discussed with the patient. Any patient who had not used the app sufficiently, according to study protocol requirements and review by the study team, was withdrawn from the study at the next scheduled appointment.

### INTELLIN^®^ acceptability ratings

Patients and clinic staff completed a questionnaire to assess aspects of platform usability on a 5-point scale (strongly agree–5, to strongly disagree–1) at Weeks 1, 16, and 52 (Supplementary Tables 2A and 2B for patients and clinicians, respectively). The questionnaire also asked whether patients and staff would recommend the platform and whether they would choose to continue using it after study conclusion.

### INTELLIN^®^ HEOR analysis

#### Cost-effectiveness model structure and output

**Figure 2.**
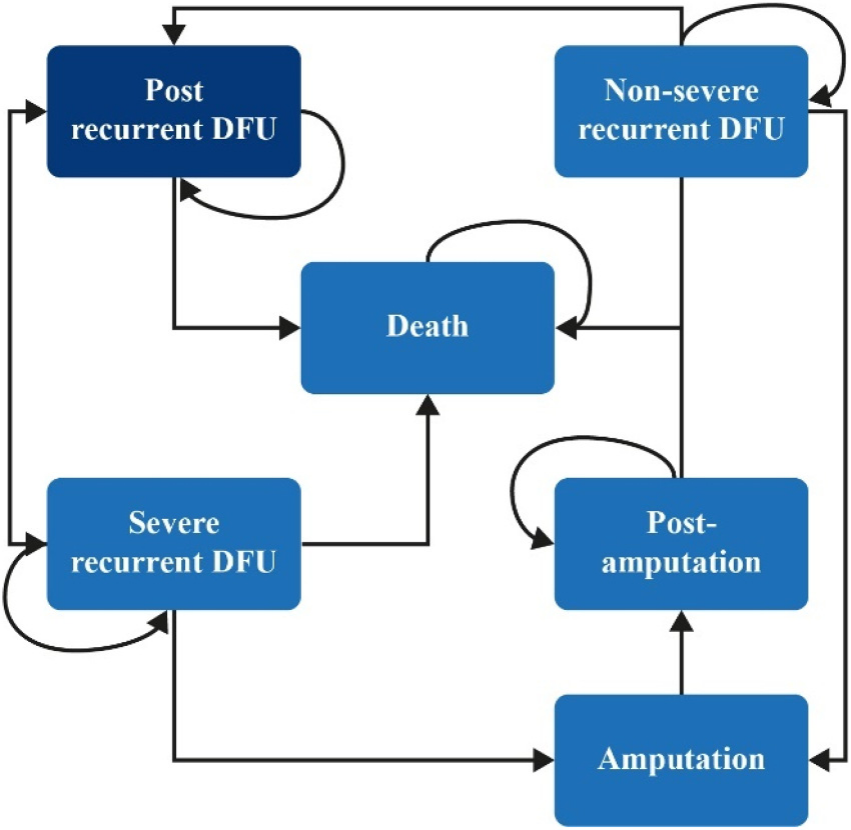
Model structure. All patients in this study started at the “post recurrent DFU” stage.

Abbreviation: DFU, diabetic foot ulcer.

A Markov cost-utility model (Figure 2) consisting of six model health states reflecting the risk of recurrent DFUs and disease progression (Table 3) was used. All patients in the hypothetical cohort had experienced a recent DFU; at baseline, 70% of patients were male and the mean age was 67 years.^16^

**Table 3.** Health states and health state utilities.

Health state	Description	Base case utility value	Reference for utility value
Post-DFU	Has had a DFU which has healed in the past 4–12 weeks	0.64	Siersma et al.^26^
Severe DFU	Has had a DFU with a SINBAD score of ≥3	0.47	Sullivan et al.^27^
Non-severe DFU	Has a DFU with a SINBAD score <3	0.61	Sullivan et al.^27^
Amputation	Receiving an amputation procedure and perioperative care	0.34	Morgan et al.^28^
Post-amputation	Receiving care and incurring costs following an amputation	0.47	Alva et al.^29^

Utilities were measured in EuroQoL-5 dimensions. Utility value for death was zero by default.

Abbreviations: DFU, diabetic foot ulcer; SINBAD, Site, Ischemia, Neuropathy, Bacterial Infection, and Depth.

In each Markov cycle of 1-month duration:

- “Post-DFU” patients could remain in the “post-DFU” category, experience a non-severe DFU (SINBAD score <3^17^) or severe DFU (SINBAD score ≥3), or die of causes unrelated to the DFU.
- Patients who experienced a non-severe DFU could remain in that category, recover, and return to the “post-DFU” category, progress to a severe DFU, or progress to an amputation.
- Patients who experienced a severe DFU could remain in that category, recover, and return to the “post-DFU” category, or further progress to amputation or death.
- “Post-amputation” patients could remain in this category or progress to death.

The model was used to predict quality-adjusted life-years (QALYs) lived and costs to calculate an incremental cost effectiveness ratio (ICER). The ICER indicates the level of benefit the INTELLIN^®^ app would need to deliver in terms of prevention of DFU recurrence to achieve cost-effectiveness at a conventional threshold for a public health intervention in the UK. Threshold analysis was conducted to provide the key output of the model, given the limited data available to fully populate the model—this reflected the lack of comparative data available for the INTELLIN^®^ app and SoC. One-way sensitivity analysis results were displayed in a tornado diagram.

#### Model clinical effectiveness measurement

Transition probabilities were obtained from the NDFA where possible. However, NDFA data was less applicable for the rate of DFU recurrence and so data for this factor was sourced from Apelqvist et al. due to its suitability for the model structure and cycle length.^18^ Mortality probabilities were obtained from Ragnarson et al., where data aligned to the health states used in this model.^19^ Lifetime time horizon was used to reflect the chronic nature of diabetes and is consistent with the NICE standard approach.^20, 21^

#### Model costs measurement

A UK NHS and Personal Social Services (PSS) perspective was adopted for the measurement of costs. Costs included in the model (Table 4) were treatment costs associated with the INTELLIN^®^ platform and health state costs, both inpatient and outpatient, associated with DFU care. The base-case INTELLIN^®^ platform cost was the monthly pricing scenario. For this analysis, an annual discount rate of 3.5% was used for future costs and outcomes, in line with NICE guidance.^21^ For severe and non-severe DFU states, a US claims data study stratifying costs by DFU severity in terms of the Wagner grading system was used for inpatient costs.^22^ It was assumed that 50% of Grade 2 patients were severe and 50% were non-severe, as no mapping between the Wagner grading and SINBAD score was found. All costs were valued in 2017/18 Great British Pounds and inflated as required using PSS Research Unit 2018.^23^

**Table 4.** Cost parameters.

Parameter	Base case value	Reference
INTELLIN^®^ app cost	-	-
Free pricing (£)	0.00	Data on file
Monthly pricing (£)	4.99	Data on file
Annual pricing (£)	4.17	Data on file
Monthly cost	-	-
Post-DFU (£)	180.70	Guest et al.^30^
Non-severe DFU (£)	453.83	Kerr^31^; Stockl et al.^22^
Severe DFU (£)	972.30	Kerr^31^; Stockl et al.^22^
Amputation (£)	1106.19	Alva et al.^32^
Post-amputation (£)	311.96	Alva et al.^32^
Diabetes app compliance	90%	Assumption

Abbreviation: DFU, diabetic foot ulcer.

## Results

### INTELLIN^®^ feasibility study results

One hundred and ninety-seven patients were assessed for eligibility and 15 entered the full analysis set (FAS).

Baseline characteristics can be seen in Table 5. At baseline, 3/15 FAS patients were female, with a mean (standard deviation [SD]) age of 60.8 (9.3) years and a mean (SD) BMI of 32.94 (6.13). All patients described their ethnicity as English. 9/15 patients previously smoked, with only one patient continuing to smoke during the study period. Only 2/15 patients completed the full study with the largest reason for incompletion being reulceration 6/15.

**Table 5.** Baseline characteristics.

Study ID	Gender	Age	Ethnicity	BMI	Employment	Smoking	Type of diabetes	Year diagnosis	Neuro loss of 10g monofilament	SINBAD score	DFU study visits	Reason for incompletion	GP visits	Out patient visits	Hospital (bed days)
001	M	75	English	37.6	Retired	Previous	2	1992	Both	2	2	Reulceration	0	0	0
002	M	72	English	29.1	Retired	Previous	1	1996	Both	2	7		4	3	3
004	M	68	English	37.2	Retired	Never	2	2006	Both	4	3	Reulceration	1	0	0
005	M	62	English	35.6	Full time	Previous	2	1997	Both	2	5	Lost to FU	4	0	0
009	F	49	English	31	Full time	Never	2	2008	None	2	7		1	8	0
010	F	53	English	31	Full time	Previous	1	1985	None	2	6	Lost to FU	1	4	0
011	M	70	English	34.2	Retired	Current	2	2017	Right	1	3	Withdrew	1	0	0
012	M	73	English	24.1	Retired	Never	2	2016	Both	2	5	Reulceration	1	1	0
013	M	61	English	45.6	Benefits	Previous	2	2007	Both	1	3	Reulceration	0	0	0
014	M	51	English	44.3	Full time	Never	2	2007	Both	1	3	Reulceration	1	0	0
015	M	66	English	29.1	Retired	Previous	2	1980	Both	1	1	Withdrew	0	0	0
016	M	51	English	27.7	Full time	Never	2	2004	None	3	3	Withdrew	0	0	0
017	M	56	English	27.6	Full time	Previous	2	2007	Both	1	4	Lost to FU	1	0	0
018	M	51	English	29.7	Benefits	Previous	1	2000	Both	4	3	Withdrew	0	0	0
019	F	54	English	30.3	Full time	Previous	2	2004	Both	3	3	Reulceration	0	0	0

Abbreviations: DFU, diabetic foot ulcer; SD, standard deviation; SINBAD, Site, Ischaemia, Neuropathy, Bacterial infection, and Depth; T1/2DM, type 1/2 diabetes mellitus.

Further baseline characteristics are summarized in Table 1. Up to Week 52, 8/15 patients in the FAS experienced a recurrence, with a mean (SD) SINBAD score of 2.1 (1.0) and a mean duration of 2.6 days. Mean time from last DFU to recurrence during follow-up was 273.0 days (95% CI 74.0, 484.0). No patients self-referred during the study.

### INTELLIN^®^ acceptability ratings

**Figure 3.**
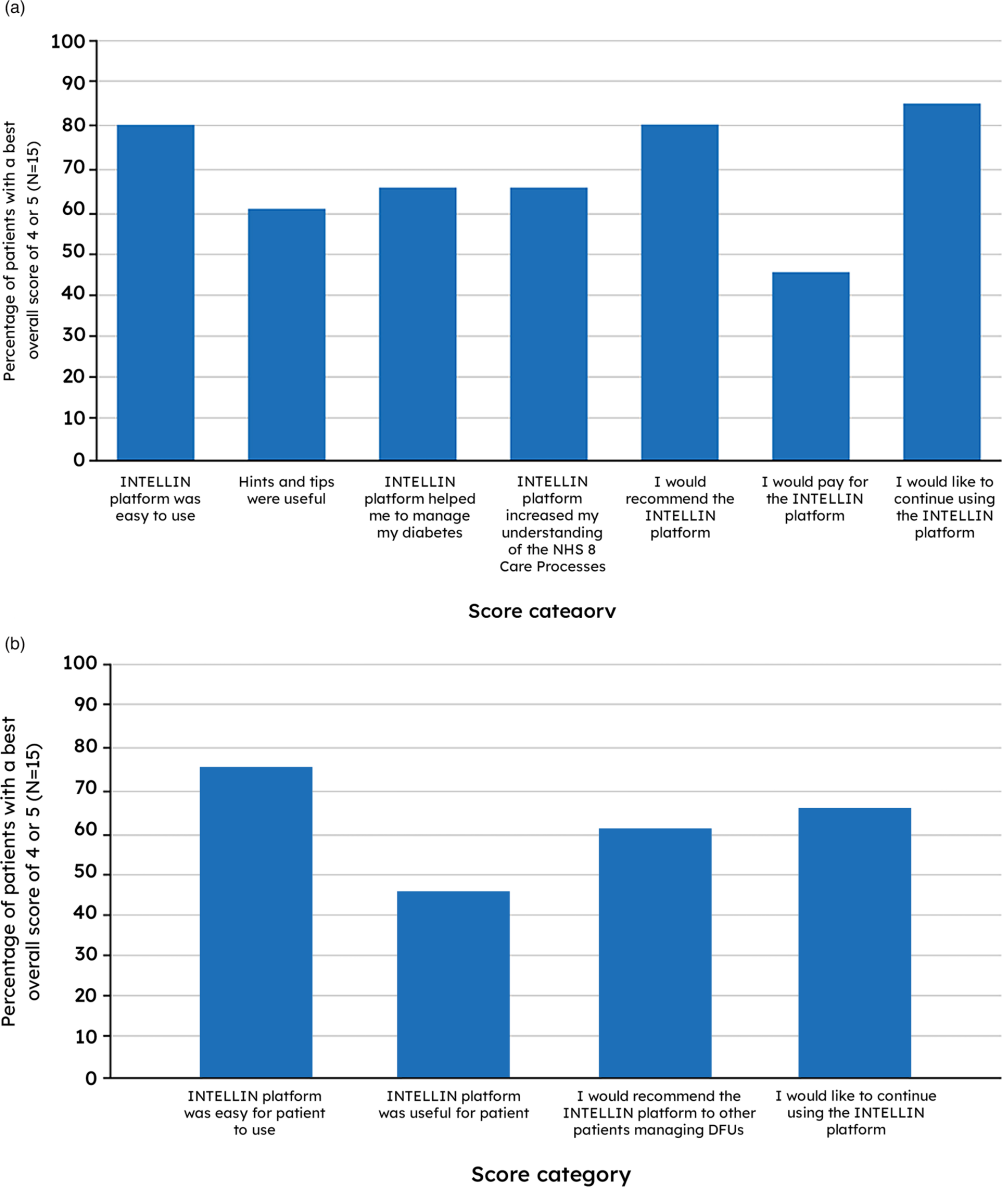
Proportion of best overall scores of 4 or 5 on the 5-point rating scale—patient (a) and clinician (b) full analysis sets.

Abbreviation: DFU, diabetic foot ulcer.

As not every study participant scored at each visit, the best overall score across visits was taken for each patient or staff member. In the qualitative assessment, 12/15 patients and 11/15 staff rated the platform 4–5/5 for ease of use overall, and 13/15 patients and 10/15 staff gave a rating of 4–5/5 for likelihood of continuing the platform after the study overall (Figure 3(a) and (b); see Supplementary Tables 3A and 3B for full summary).

### Socio-economic impact on study recruitment

Reasons underlying enrollment failure are summarized in Table 6. App accessibility issues predominated (69/178; 38.8%), driven by a lack of smartphone access (58/178; 32.6%), which was the most important barrier to enrollment, with subsequent patient discussion highlighting concerns over use of mobile data packages and phone memory. Eyesight problems (5/178 patients; 2.8%) and cognitive impairment (3/178 patients; 1.7%) were also identified as barriers to smartphone use and/or enrollment. Medical/compliance issues (63/178; 35.4%) and personal reasons for non-participation (46/178; 25.8%) were identified as additional major barriers. Of those patients that entered the FAS, two completed the study.

**Table 6.** Reasons for patient non-participation in the study.

Reason for non-participation	Patients (*N* = 178), *n* (%)
App accessibility issues	69 (38.8)
Language	3 (1.7)
Cognitive impairment	3 (1.7)
Eyesight *(limiting use of smartphone)*	5 (2.8)
No smartphone	58 (32.6)
Medical/compliance issues	63 (35.4)
Reulcerated prior to study commencement	28 (15.7)
Deceased	2 (1.1)
Awaiting other surgery *(not necessarily foot or diabetes related)*	2 (1.1)
Outside 12-week rule *(history of a recent DFU defined as eligible for the NDFA and classified as healed for ≥4 weeks but no more than 12 weeks)*	20 (11.2)
Future appointments already planned *(not necessarily foot or diabetes related)*	4 (2.2)
In another study	2 (1.1)
Compliance issues *(patients unlikely to be able to attend all visits necessary for study inclusion)*	5 (2.8)
Personal issues	46 (25.8)
No interest	9 (5.0)
No contact	24 (13.5)
Moved area	3 (1.7)
Domiciliary *(patients requiring home-based care)*	6 (3.4)
Work	3 (1.7)
Personal reasons (other)	1 (0.6)

Abbreviations: DFU, diabetic foot ulcer; NDFA, National Diabetes Foot Care Audit.

### INTELLIN^®^ HEOR analysis results

**Figure 4.**
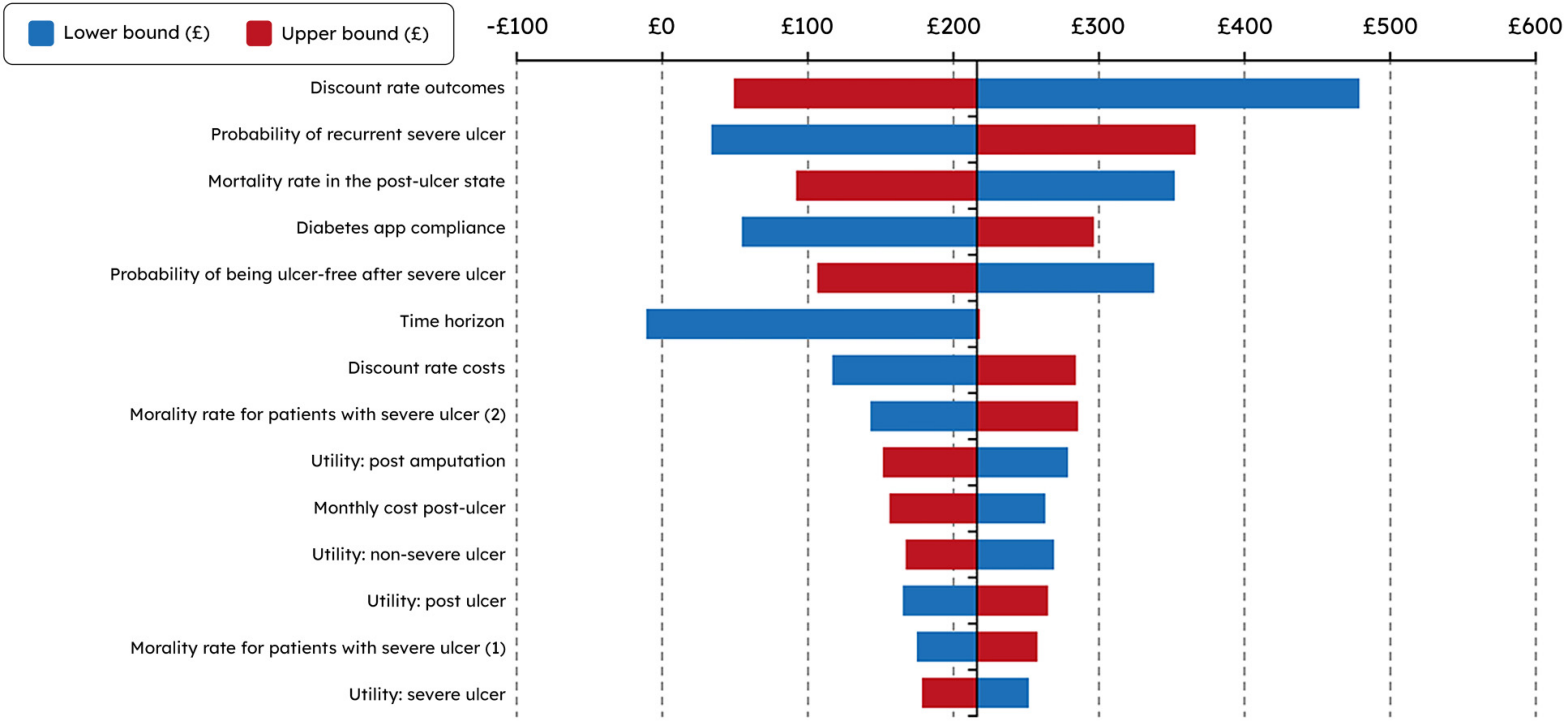
Results from the one-way sensitivity analysis for the base case represented graphically in a tornado diagram showing variation in the net monetary benefit.

Note: This figure shows the one-way sensitivity analysis result when the threshold analysis for relative risk was run with the base case settings and the resulting relative risk inputted in the model, giving an ICER of £20,000 and net monetary benefit of £215.48. Abbreviation: ICER, incremental cost effectiveness ratio.

The model demonstrated that the platform required a relative reduction in DFU recurrence of only 3% (relative risk 0.97) compared with SoC to attain an ICER of £20,000 per QALY. One-way sensitivity analysis showed that the discount rate for outcomes, mortality rate in the post-ulcer state, and probability of recurrent severe ulcers were the factors with the greatest impact on the outcomes of the model (Figure 4).

When mortality data were sourced from the NDFA 2015–2018,^4^ the platform required a relative reduction in DFU recurrence of 5% (relative risk 0.95) compared with SoC to attain an ICER of £20,000 per QALY. In a scenario in which the cost of the platform was reduced to £4.17 on average per month, according to an annual pricing schedule, the platform required a relative reduction in DFU recurrence of 3% (relative risk 0.97) compared with SoC to attain an ICER of £20,000 per QALY.

## Discussion

### Analysis of other DFU solutions

There are mobile apps specifically for DFU management to aid healthcare professionals diagnose and monitor progression of DFU (DFUAPP; FootSnap). Patient facing apps seem to concentrate on either education (MyFootCare) or trackers (Signs & Symptoms Diabetic Foot).

There are more than 1000 diabetes mobile applications available on the Apple App Store and Goggle Play; however, none have the clinician dashboard interface that connects to the electronic health records facilitating remote monitoring and proactive care like INTELLIN^^®^^. Many like MySugr and Glucose Buddy are tracking apps which allow people to monitor and track their blood glucose or carbohydrate intake. INTELLIN^^®^^ goes beyond this linking to other devices tracking is made easier, the addition of focused clinically approved information along with the connectivity to the healthcare professionals dashboard makes INTELLIN^^®^^ unique.

### Analysis of feasibility study results

The number of ulcer-free days reported in this feasibility study (mean 273.0 days) is high compared to previous reports (median 233 days).^24^ There were no instances of self-referral for a DFU reported, although this finding is likely to be associated with the prospective visits already scheduled within the study and patient familiarity with the emergency appointment service provided by SRFT. Despite the small sample size, initial qualitative data shows high INTELLIN^®^ platform acceptability and usability for patients and clinicians, with high likelihood of recommendation for future use. Future studies may explore the potential for increased time to reulceration compared with published data.

### Analysis of INTELLIN^®^ cost-effectiveness

The factors with the greatest impact on the model for ICER in a comparison of INTELLIN^®^ with SoC were the discount rate for outcomes, mortality rate in the post-ulcer state, and probability of recurrent severe DFUs. The latter is consistent with the finding that severe DFUs are associated with the greatest cost, and once patients experience a severe recurrent DFU, their chance of that DFU resolving is lower than that with a non-severe recurrent DFU.^25^

In this model, the INTELLIN^®^ platform only required a relative reduction in DFU recurrence of 5% compared with SoC to attain an ICER of £20,000 per QALY. Furthermore, in the scenario in which the average monthly app cost was reduced to £4.17, the app required a relative reduction in DFU recurrence of just 3% compared with SoC to attain an ICER of £20,000 per QALY. These values suggest the potential for significant healthcare cost savings upon wider adoption of the INTELLIN^®^ platform, alongside SoC, within the healthcare system.

### Analysis of study recruitment barriers

Data taken from the NDFA identified that SRFT has ∼32 new episodes of ulceration a month and approximately 58% of SRFT patients are alive and ulcer free at 12 weeks.^13^ This indicated there could potentially be 15–18 people every month eligible for inclusion in the study.

The substantial barriers to enrollment encountered in this feasibility study demonstrate the considerable impact of socio-economic factors on mHealth studies, specifically in this study in patients with diabetes. The diabetes demographic often includes older patients and those with comorbidities, which may also result in a high burden of technology accessibility issues. These issues may have been compounded by the relatively low-income demographic of the Salford ward.

## Conclusions

The results of this feasibility and accessibility study combined with an HEOR evaluation suggest that the INTELLIN^®^ platform needs only to provide a small reduction in DFU recurrence compared to SoC to be a cost-effective strategy for the prevention of recurrent DFUs. This is a promising result and supports the future development of a comparative study to evaluate the effectiveness of the platform compared with SoC in patients with complications due to type 1 or type 2 diabetes.

Despite the small sample size, initial qualitative data shows high INTELLIN^®^ platform acceptability and usability for patients and clinicians, with high likelihood of recommendation for future use. The number of ulcer-free days reported in this study is high compared to previous reports. Future studies will be warranted to explore the potential for increased time to reulceration versus published data. Future studies should also consider appropriate measures and additional support required for increased study accessibility.

## Supplemental Material

sj-docx-1-dhj-10.1177_20552076221142103 - Supplemental material for Implementation of a novel mHealth application for the management of people with diabetes and recently healed foot ulceration: A feasibility study Click here for additional data file. Supplemental material, sj-docx-1-dhj-10.1177_20552076221142103 for Implementation of a novel mHealth application for the management of people with diabetes and recently healed foot ulceration: A feasibility study by Samantha Haycocks, Rory Cameron, Mark Edge, Jayne Budd and Paul Chadwick in Digital Health

## Abbreviations

BMI: body mass index
CI: confidence interval
DFU: diabetic foot ulcer
FAS: full analysis set
HbA_1c_: glycated hemoglobin
HEOR: health economics and outcomes research
ICER: incremental cost-effectiveness ratio
NDFA: National Diabetes Footcare Audit
NHS: National Health Service (UK)
NICE: National Institute for Health and Care Excellence
PSS: Personal Social Services
QALY: quality-adjusted life-years
SD: standard deviation
SINBAD: Site, Ischemia, Neuropathy, Bacterial Infection, and Depth
SoC: standard of care
SRFT: Salford Royal NHS Foundation Trust
