# Supplementary material for: Implementation of a novel mHealth application for the management of people with diabetes and recently healed foot ulceration: A feasibility study
Source: Digit Health. 2022 Dec 6;8:20552076221142103. doi: 10.1177/20552076221142103 (PMC9732796; doi:10.1177/20552076221142103)
Supplement: sj-docx-1-dhj-10.1177_20552076221142103 - Supplemental material for Implementation of a novel mHealth application for the management of people with diabetes and recently healed foot ulceration: A feasibility study [file sj-docx-1-dhj-10.1177_20552076221142103.docx]

## Supplemental Material for Implementation of a Novel mHealth Application for the Management of People with Diabetes and Recently Healed Foot Ulceration: A Feasibility Study

**Authors:** Samantha Haycocks^1*^, Rory Cameron^2^, Mark Edge^2^, Jayne Budd^2^, Paul Chadwick^3*^

^*^Samantha Haycocks and Paul Chadwick contributed equally to this paper.

^1^Salford Care Organisation, Northern Care Alliance NHS Foundation Trust, Salford, UK.

^2^Gendius Ltd, Macclesfield, UK.

^3^Royal College of Podiatry, London, UK.

## Supplementary Data

**Supplemental Table 1.** App Profile Questions.

| **Questions in the front end of the app used at first log in:** |
| --- |
| 1. How would you like to be known by INTELLIN? |
| 2. What type of diabetes do you have? |
| 3. What gender are you? (*Options MALE/FEMALE/PREFER NOT TO SAY*) |
| 4. What are the first 4 digits of your postcode? |
| 5. What year were you born? |
| 6. What is your ethnicity? (*Option to PREFER NOT TO SAY*) |
| 7. What is your height? |
| 8. What is your weight? |
| 9. Do you smoke? |
| 10. What is your average weekly alcohol intake? (1 unit=1 shot, 1/3 pint or 1/2 standard glass of wine) |
| 11. What is the intensity level of any exercise you do? |
| 12. How long do you usually exercise for every week? |
| 13. What year were you diagnosed with diabetes? (this calculates how long you have had diabetes) |
| 14. What is your average daily basal insulin dose? |
| 15. What is your average daily bolus insulin dose? |
| 16. What was your last blood glucose reading? |
| 17. Do you take medication to manage your cholesterol levels? |
| 18. Do you take medication to control your blood pressure? |
| 19. Do you currently have, or have you had, atrial fibrillation? |
| 20. Do you currently have, or have you had, a foot deformity or callus? |
| 21. Do you currently have, or have you had, a diabetic foot ulcer? |
| 22. Do you currently have, or have you had, problems with your eyes? |
| 23. Do you currently have, or have you had, problems with your kidneys? |
| 24. Do you currently have, or have you had, sensation loss in your feet? |
| 25. Have you ever had to have help from anyone to recover from a hypo (low blood sugar)? |
| 26. How many times have you had a diabetes-related emergency admission to hospital in the last 5 years? |
| 27. What was your last urinary albumin reading? |
| 28. What was your last serum creatinine reading? |
| 29. What was your last HbA_1c_ reading? |
| 30. What was your last systolic blood pressure reading? |
| 31. What was your last diastolic blood pressure reading? |
| 32. What was your last triglyceride reading? |
| 33. What was your last total cholesterol reading? |
| 34. What was your last HDL reading? |
| 35. What was your last LDL reading? |
| 36. What was your last retinopathy/eye test reading? |

**Abbreviations:** HbA_1c_, glycated hemoglobin; HDL, high-density lipoprotein; LDL, low-density lipoprotein.

**Supplemental Table 2A**. INTELLIN User Experience Questionnaire.

|  | | **Strongly disagree** | **Disagree** | **Neither agree nor disagree** | **Agree** | **Strongly Agree** |
| --- | --- | --- | --- | --- | --- | --- |
|  | **Question** | **1** | **2** | **3** | **4** | **5** |
| 1. | The INTELLIN platform was easy to use |  |  |  |  |  |
| 2. | The hints and tips were useful |  |  |  |  |  |
| 3. | The INTELLIN platform helped me to manage my diabetes |  |  |  |  |  |
| 4. | The INTELLIN app increased my understanding of the 8 annual checks and how they impact on my diabetes |  |  |  |  |  |
| 5. | I would recommend the INTELLIN platform to someone else who has had a diabetic foot ulcer |  |  |  |  |  |
| 6. | I would pay for the INTELLIN platform as a way of helping manage my feet/diabetes |  |  |  |  |  |
| 7. | I would like to continue to use the platform after the study |  |  |  |  |  |

Any other comments/suggestions?

**Supplemental Table 2B.** INTELLIN Clinician Experience Questionnaire.

|  |  | **Strongly disagree** | **Disagree** | **Neither agree nor disagree** | **Agree** | **Strongly Agree** |
| --- | --- | --- | --- | --- | --- | --- |
|  | **Question** | **1** | **2** | **3** | **4** | **5** |
| 1. | The INTELLIN platform was easy for my patients to use |  |  |  |  |  |
| 2. | The INTELLIN platform was useful for my patients |  |  |  |  |  |
| 3. | I would recommend the INTELLIN platform to someone else managing people with diabetic foot ulcers |  |  |  |  |  |
| 4. | I would like to continue to offer the INTELLIN platform to patients after the study |  |  |  |  |  |

Any other comments/suggestions?

**Supplemental Table 3A.** Summary of INTELLIN Patient Experience Questionnaire Results on a 5-Point Rating Scale (Full Analysis Set, *N* = 15).

|  | | **5-point rating score** | | | | | | |
| --- | --- | --- | --- | --- | --- | --- | --- | --- |
| **Patient app rating, *n* (%)** | | **1** | **2** | **3** | **4** | **5** | **4 or 5** | **Missing** |
| **The INTELLIN platform was easy to**  **use** | **Visit 1 (baseline)** | 0 | 0 | 0 | 1  (6.7) | 1  (6.7) | 2 (13.3) | 13 (86.7) |
|  | **Visit 2 (Week 1)** | 0 | 1  (6.7) | 2  (13.3) | 7  (46.7) | 4 (26.7) | 11 (73.3) | 0 |
|  | **Visit 3 (Week 4)** | 0 | 0 | 1  (6.7) | 1  (6.7) | 1  (6.7) | 2 (13.3) | 7  (46.7) |
|  | **Visit 4 (Week 16)** | 0 | 0 | 0 | 4  (26.7) | 2 (13.3) | 6 (40.0) | 0 |
|  | **Visit 5 (Week 28)** | 0 | 0 | 0 | 0 | 1  (6.7) | 1  (6.7) | 3  (20.0) |
|  | **Visit 6 (Week 40)** | 0 | 0 | 0 | 2  (13.3) | 0 | 2 (13.3) | 1 (6.7) |
|  | **Visit 7 (Week 52)** | 0 | 0 | 0 | 2  (13.3) | 0 | 2 (13.3) | 0 |
|  | **Visit unknown** | 0 | 0 | 0 | 0 | 0 | 0 | 9  (60.0) |
|  | **Best overall**  **score** | 0 | 0 | 3  (20.0) | 7  (46.7) | 5 (33.3) | 12 (80.0) | 0 |
| **The hints and tips were useful** | **Visit 1 (baseline)** | 0 | 0 | 0 | 0 | 0 | 0 | 15 (100) |
|  | **Visit 2 (Week 1)** | 1  (6.7) | 0 | 1  (6.7) | 4  (26.7) | 4 (26.7) | 8 (53.3) | 4  (26.7) |
|  | **Visit 3 (Week 4)** | 0 | 0 | 0 | 1  (6.7) | 1  (6.7) | 2 (13.3) | 8  (53.3) |
|  | **Visit 4 (Week 16)** | 0 | 0 | 1  (6.7) | 2  (13.3) | 2 (13.3) | 4 (26.7) | 1  (6.7) |
|  | **Visit 5 (Week 28)** | 0 | 0 | 0 | 0 | 1  (6.7) | 1  (6.7) | 3  (20.0) |
|  | **Visit 6 (Week 40)** | 0 | 0 | 1  (6.7) | 0 | 1  (6.7) | 1  (6.7) | 1  (6.7) |
|  | **Visit 7 (Week 52)** | 0 | 0 | 1  (6.7) | 0 | 1  (6.7) | 1  (6.7) | 0 |
|  | **Visit unknown** | 0 | 0 | 0 | 0 | 0 | 0 | 9  (60.0) |
|  | **Best overall**  **score** | 1  (6.7) | 0 | 1  (6.7) | 5  (33.3) | 4 (26.7) | 9 (60.0) | 4  (26.7) |
| **The INTELLIN platform helped me**  **to manage my diabetes** | **Visit 1 (baseline)** | 0 | 0 | 0 | 1  (6.7) | 1  (6.7) | 2 (13.3) | 13 (86.7) |
|  | **Visit 2 (Week 1)** | 0 | 0 | 5  (33.3) | 4  (26.7) | 3 (20.0) | 7 (46.7) | 2  (13.3) |
|  | **Visit 3 (Week 4)** | 0 | 0 | 0 | 2  (13.3) | 1  (6.7) | 3 (20.0) | 7  (46.7) |
|  | **Visit 4 (Week 16)** | 0 | 1  (6.7) | 1  (6.7) | 2  (13.3) | 2 (13.3) | 4 (26.7) | 0 |
|  | **Visit 5 (Week 28)** | 0 | 0 | 0 | 0 | 1  (6.7) | 1  (6.7) | 3  (20.0) |
|  | **Visit 6 (Week 40)** | 0 | 1  (6.7) | 0 | 0 | 1  (6.7) | 1  (6.7) | 1  (6.7) |
|  | **Visit 7 (Week 52)** | 0 | 1  (6.7) | 0 | 0 | 1  (6.7) | 1  (6.7) | 0 |
|  | **Visit unknown** | 0 | 0 | 0 | 0 | 0 | 0 | 9  (60.0) |
|  | **Best overall**  **score** | 0 | 0 | 4  (26.7) | 6  (40.0) | 4 (26.7) | 10 (66.7) | 1  (6.7) |
| **The INTELLIN app increased my**  **understanding of the 8 Care Processes and how they impact on my**  **diabetes** | **Visit 1 (baseline)** | 0 | 1  (6.7) | 0 | 0 | 1  (6.7) | 1  (6.7) | 13 (86.7) |
|  | **Visit 2 (Week 1)** | 1  (6.7) | 0 | 3  (20.0) | 3  (20.0) | 5 (33.3) | 8 (53.3) | 2  (13.3) |
|  | **Visit 3 (Week 4)** | 0 | 0 | 0 | 2  (13.3) | 1  (6.7) | 3 (20.0) | 7  (46.7) |
|  | **Visit 4 (Week 16)** | 0 | 0 | 1  (6.7) | 4  (26.7) | 1  (6.7) | 5 (33.3) | 0 |
|  | **Visit 5 (Week 28)** | 0 | 0 | 0 | 0 | 1  (6.7) | 1  (6.7) | 3  (20.0) |
|  | **Visit 6 (Week 40)** | 0 | 1  (6.7) | 0 | 0 | 1  (6.7) | 1  (6.7) | 1  (6.7) |
|  | **Visit 7 (Week 52)** | 0 | 1  (6.7) | 0 | 0 | 1  (6.7) | 1  (6.7) | 0 |
|  | **Visit unknown** | 0 | 0 | 0 | 0 | 0 | 0 | 9  (60.0) |
|  | **Best overall**  **score** | 0 | 1  (6.7) | 3  (20.0) | 5  (33.3) | 5 (33.3) | 10 (66.7) | 1  (6.7) |
| **I would recommend the INTELLIN**  **platform to someone else who has**  **had a diabetic foot ulcer** | **Visit 1 (baseline)** | 0 | 0 | 0 | 1  (6.7) | 1  (6.7) | 2 (13.3) | 13 (86.7) |
|  | **Visit 2 (Week 1)** | 1  (6.7) | 0 | 2  (13.3) | 2  (13.3) | 7 (46.7) | 9 (60.0) | 2  (13.3) |
|  | **Visit 3 (Week 4)** | 0 | 0 | 0 | 2  (13.3) | 1  (6.7) | 3 (20.0) | 7  (46.7) |
|  | **Visit 4 (Week 16)** | 0 | 0 | 0 | 3  (20.0) | 3 (20.0) | 6 (40.0) | 0 |
|  | **Visit 5 (Week 28)** | 0 | 0 | 0 | 0 | 1  (6.7) | 1  (6.7) | 3  (20.0) |
|  | **Visit 6 (Week 40)** | 0 | 0 | 0 | 1  (6.7) | 1  (6.7) | 2 (13.3) | 1  (6.7) |
|  | **Visit 7 (Week 52)** | 0 | 0 | 0 | 1  (6.7) | 1  (6.7) | 2 (13.3) | 0 |
|  | **Visit unknown** | 0 | 0 | 0 | 0 | 0 | 0 | 9  (60.0) |
|  | **Best overall**  **score** | 0 | 0 | 2  (13.3) | 5  (33.3) | 7 (46.7) | 12 (80.0) | 1  (6.7) |
| **I would pay for the INTELLIN**  **platform as a way of helping**  **manage my feet/diabetes** | **Visit 1 (baseline)** | 0 | 0 | 1  (6.7) | 0 | 1  (6.7) | 1  (6.7) | 13 (86.7) |
|  | **Visit 2 (Week 1)** | 1  (6.7) | 4  (26.7) | 3  (20.0) | 1  (6.7) | 4 (26.7) | 5 (33.3) | 1  (6.7) |
|  | **Visit 3 (Week 4)** | 0 | 0 | 1  (6.7) | 1  (6.7) | 1  (6.7) | 2 (13.3) | 7  (46.7) |
|  | **Visit 4 (Week 16)** | 3 (20.0) | 0 | 0 | 1  (6.7) | 2 (13.3) | 3 (20.0) | 0 |
|  | **Visit 5 (Week 28)** | 0 | 0 | 0 | 0 | 1  (6.7) | 1  (6.7) | 3  (20.0) |
|  | **Visit 6 (Week 40)** | 1  (6.7) | 0 | 0 | 0 | 1  (6.7) | 1  (6.7) | 1  (6.7) |
|  | **Visit 7 (Week 52)** | 1  (6.7) | 0 | 0 | 0 | 1  (6.7) | 1  (6.7) | 0 |
|  | **Visit unknown** | 0 | 0 | 0 | 0 | 0 | 0 | 9  (60.0) |
|  | **Best overall**  **score** | 1  (6.7) | 3  (20.0) | 4  (26.7) | 3  (20.0) | 4 (26.7) | 7 (46.7) | 0 |
| **I would like to continue to use**  **the platform after the study** | **Visit 1 (baseline)** | 0 | 0 | 1  (6.7) | 0 | 1  (6.7) | 1  (6.7) | 13 (86.7) |
|  | **Visit 2 (Week 1)** | 1  (6.7) | 0 | 3  (20.0) | 3  (20.0) | 6 (40.0) | 9 (60.0) | 1  (6.7) |
|  | **Visit 3 (Week 4)** | 0 | 0 | 0 | 2  (13.3) | 1  (6.7) | 3 (20.0) | 7  (46.7) |
|  | **Visit 4 (Week 16)** | 0 | 0 | 1  (6.7) | 3  (20.0) | 2 (13.3) | 5 (33.3) | 0 |
|  | **Visit 5 (Week 28)** | 0 | 0 | 0 | 0 | 1  (6.7) | 1  (6.7) | 3  (20.0) |
|  | **Visit 6 (Week 40)** | 0 | 0 | 1  (6.7) | 0 | 1  (6.7) | 1  (6.7) | 1  (6.7) |
|  | **Visit 7 (Week 52)** | 0 | 0 | 1  (6.7) | 0 | 1  (6.7) | 1  (6.7) | 0 |
|  | **Visit unknown** | 0 | 0 | 0 | 0 | 0 | 0 | 9  (60.0) |
|  | **Best overall**  **score** | 0 | 0 | 2  (13.3) | 7  (46.7) | 6 (40.0) | 13 (86.7) | 0 |

Percentages are calculated using the number of patients in the full analysis set as the denominator.

Missing is derived as the number of patients who have no 5-point rating score, who are still in the study at that timepoint. 1: Strongly disagree; 2: Disagree; 3: Neither agree nor disagree; 4: Agree; 5: Strongly agree.

**Supplemental Table 3B**. Summary of INTELLIN clinician experience questionnaire results on a 5‑point rating scale (full analysis set, *N* = 15).

|  | | **5-point rating score** | | | | | | |
| --- | --- | --- | --- | --- | --- | --- | --- | --- |
| **Clinician app rating, n (%)** | | **1** | **2** | **3** | **4** | **5** | **4 or 5** | **Missing** |
| **The INTELLIN platform was easy for my patients to use** | **Visit 1 (baseline)** | 0 | 0 | 0 | 2  (13.3) | 0 | 2 (13.3) | 13 (86.7) |
|  | **Visit 2 (Week 1)** | 1  (6.7) | 2  (13.3) | 2  (13.3) | 6  (53.3) | 1  (6.7) | 9 (60.0) | 0 |
|  | **Visit 3 (Week 4)** | 1  (6.7) | 0 | 0 | 1  (6.7) | 0 | 1  (6.7) | 6  (53.3) |
|  | **Visit 4 (Week 16)** | 0 | 0 | 1  (6.7) | 4  (26.7) | 1  (6.7) | 5 (33.3) | 0 |
|  | **Visit 5 (Week 28)** | 0 | 0 | 0 | 0 | 0 | 0 | 4  (46.7) |
|  | **Visit 6 (Week 40)** | 0 | 0 | 1  (6.7) | 1  (6.7) | 0 | 1  (6.7) | 1  (6.7) |
|  | **Visit 7 (Week 52)** | 0 | 0 | 0 | 1  (6.7) | 1  (6.7) | 2 (13.3) | 0 |
|  | **Visit unknown** | 0 | 0 | 0 | 0 | 0 | 0 | 9  (60.0) |
|  | **Best overall**  **score** | 1  (6.7) | 1  (6.7) | 2  (13.3) | 6  (53.3) | 3 (20.0) | 11 (73.3) | 0 |
| **The INTELLIN platform was useful for my patients** | **Visit 1 (baseline)** | 0 | 0 | 2  (13.3) | 0 | 0 | 0 | 13 (86.7) |
|  | **Visit 2 (Week 1)** | 1  (6.7) | 2  (13.3) | 5  (33.3) | 5  (33.3) | 0 | 5 (33.3) | 1  (6.7) |
|  | **Visit 3 (Week 4)** | 0 | 1  (6.7) | 0 | 1  (6.7) | 0 | 1  (6.7) | 8  (53.3) |
|  | **Visit 4 (Week 16)** | 0 | 1  (6.7) | 1  (6.7) | 3  (20.0) | 1  (6.7) | 4 (26.7) | 0 |
|  | **Visit 5 (Week 28)** | 0 | 0 | 0 | 0 | 0 | 0 | 4  (26.7) |
|  | **Visit 6 (Week 40)** | 0 | 0 | 1  (6.7) | 1  (6.7) | 0 | 1  (6.7) | 1  (6.7) |
|  | **Visit 7 (Week 52)** | 0 | 0 | 1  (6.7) | 0 | 1  (6.7) | 1  (6.7) | 0 |
|  | **Visit unknown** | 0 | 0 | 0 | 0 | 0 | 0 | 9  (60.0) |
|  | **Best overall**  **score** | 1  (6.7) | 2  (13.3) | 5  (33.3) | 6  (40.0) | 1  (6.7) | 7 (46.7) | 0 |
| **I would recommend the INTELLIN**  **platform to someone else**  **managing people with diabetic**  **foot ulcers** | **Visit 1 (baseline)** | 0 | 0 | 1  (6.7) | 1  (6.7) | 0 | 1  (6.7) | 13 (86.7) |
|  | **Visit 2 (Week 1)** | 0 | 2  (13.3) | 3  (20.0) | 7  (46.7) | 1  (6.7) | 8 (53.3) | 1  (6.7) |
|  | **Visit 3 (Week 4)** | 0 | 0 | 1  (6.7) | 1  (6.7) | 0 | 1  (6.7) | 8  (53.3) |
|  | **Visit 4 (Week 16)** | 0 | 1  (6.7) | 1  (6.7) | 4  (26.7) | 0 | 4 (26.7) | 0 |
|  | **Visit 5 (Week 28)** | 0 | 0 | 0 | 0 | 0 | 0 | 4  (26.7) |
|  | **Visit 6 (Week 40)** | 0 | 0 | 1  (6.7) | 1  (6.7) | 0 | 1  (6.7) | 1  (6.7) |
|  | **Visit 7 (Week 52)** | 0 | 0 | 1  (6.7) | 1  (6.7) | 0 | 1  (6.7) | 0 |
|  | **Visit unknown** | 0 | 0 | 0 | 0 | 0 | 0 | 9  (60.0) |
|  | **Best overall**  **score** | 0 | 1  (6.7) | 5  (33.3) | 6  (53.3) | 1  (6.7) | 9 (60.0) | 0 |
| **I would like to continue to**  **offer the INTELLIN platform to**  **patients after the study** | **Visit 1 (baseline)** | 0 | 0 | 0 | 2  (13.3) | 0 | 2 (13.3) | 13 (86.7) |
|  | **Visit 2 (Week 1)** | 0 | 2  (13.3) | 3  (20.0) | 6  (40.0) | 2 (13.3) | 8 (53.3) | 1  (6.7) |
|  | **Visit 3 (Week 4)** | 0 | 0 | 1  (6.7) | 1  (6.7) | 0 | 1  (6.7) | 8  (53.5) |
|  | **Visit 4 (Week 16)** | 0 | 0 | 1  (6.7) | 5  (33.3) | 0 | 5 (33.3) | 0 |
|  | **Visit 5 (Week 28)** | 0 | 0 | 0 | 0 | 0 | 0 | 4  (26.7) |
|  | **Visit 6 (Week 40)** | 0 | 0 | 1  (6.7) | 1  (6.7) | 0 | 1  (6.7) | 1  (6.7) |
|  | **Visit 7 (Week 52)** | 0 | 0 | 2  (13.3) | 0 | 0 | 0 | 0 |
|  | **Visit unknown** | 0 | 0 | 0 | 0 | 0 | 0 | 9  (60.0) |
|  | **Best overall**  **score** | 0 | 1  (6.7) | 4  (26.7) | 8  (53.3) | 2 (13.3) | 10 (66.7) | 0 |

Percentages are calculated using the number of clinicians in the full analysis set as the denominator.

Missing is derived as the number of clinicians who have no 5-point rating score, who are still in the study at that timepoint. 1: Strongly disagree; 2: Disagree; 3: Neither agree nor disagree; 4: Agree; 5: Strongly agree.

**Supplementary Figure 1.** INTELLIN^®^ platform healthcare professional dashboard and patient mobile app.


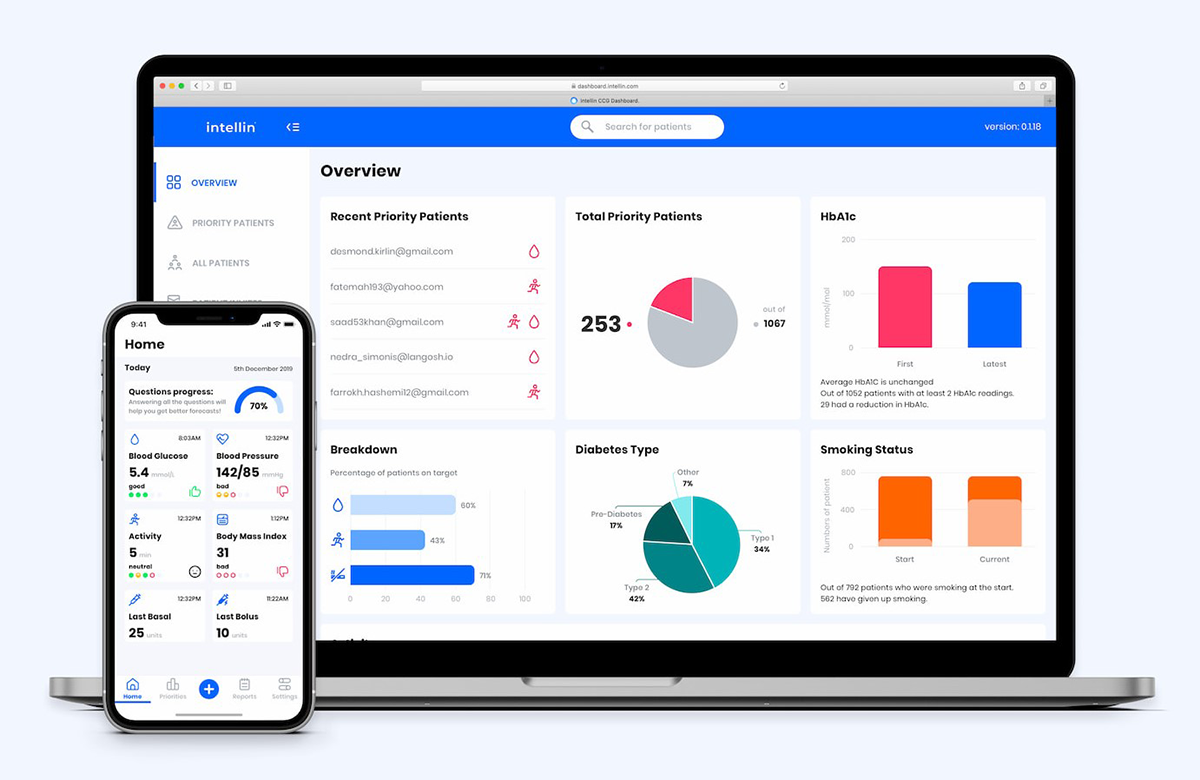


**Abbreviation:** HbA_1c_, glycated hemoglobin.
